# Efficacy of Antibiotic‐Loaded Hydroxyapatite/Collagen Composites Is Dependent on Adsorbability for Treating *Staphylococcus aureus* Osteomyelitis in Rats

**DOI:** 10.1002/jor.24507

**Published:** 2019-11-17

**Authors:** Satoru Egawa, Keigo Hirai, Rempei Matsumoto, Toshitaka Yoshii, Masato Yuasa, Atsushi Okawa, Ken Sugo, Shinichi Sotome

**Affiliations:** ^1^ Department of Orthopaedic and Spinal Surgery, Graduate School Tokyo Medical and Dental University 1‐5‐45 Yushima, Bunkyo‐ku Tokyo 113‐8519 Japan; ^2^ HOYA Technosurgical Corporation 1‐1‐110, Tsutsujigaoka Akishima‐shi Tokyo 196‐0012 Japan; ^3^ Department of Orthopaedic Research and Development, Graduate School Tokyo, Medical and Dental University 1‐5‐45 Yushima, Bunkyo‐ku Tokyo 113‐8519 Japan

**Keywords:** hydroxyapatite collagen (HAp/Col), osteomyelitis, *Staphylococcus aureus*, adsorbability, vancomycin

## Abstract

Osteomyelitis remains one of the most challenging disorders for orthopedic doctors despite the advancement of therapeutic techniques. The purpose of this study was to investigate the feasibility of local antibiotic administration using hydroxyapatite/collagen (HAp/Col) as a drug delivery system. We hypothesized that higher adsorbability of antibiotics onto HAp/Col will result in more efficacious activity and therefore, treatment of osteomyelitis. Eight antibiotics were examined in this study: amikacin, cefazolin, cefotiam, daptomycin, minocycline, piperacillin, teicoplanin, and vancomycin. Aligning with their adsorbability onto HAp/Col, minocycline, teicoplanin, and vancomycin showed antibacterial effects up to 14 days after subcutaneous implantation in Wistar rats; while antibiotics with reduced adsorbability (cefazolin, cefotiam, piperacillin) had diminished antibacterial effects. Furthermore, when implanted into a rat femur, vancomycin levels from the Hap/Col were detected in the medullary space above the minimum inhibitory concentration for *Staphylococcus aureus* for 7 days, while cefazolin levels were undetectable. Aligning with these results, implantation of Hap/Col impregnated with vancomycin to the femur in an acute osteomyelitis rat model had a greater therapeutic effect than cefazolin, as measured by the number of bacteria, the extent of bone destruction, and bone regeneration. These results indicated that the adsorbability of antibiotics onto their carrier is important when locally administered and that HAp/Col scaffolds might be a useful antibiotic delivery system for osteomyelitis. © 2019 The Authors. Journal of Orthopaedic Research® published by Wiley Periodicals, Inc. on behalf of Orthopaedic Research Society J Orthop Res 38:843‐851, 2020

## Graphical abstract

This study aimed to investigate the feasibility of local antibiotic administration using hydroxyapatite collagen (HAp/Col) composite as a drug delivery system. Minomycin, teicoplanin, and vancomycin showed antibacterial effects up to 14 days after subcutaneous implantation in rats with HAp/Col owing to their adsorbability. The implantation of HAp/Col impregnated with vancomycin for an acute osteomyelitis model revealed a significant therapeutic effect because of longitudinal antibacterial activity. These results indicated that the adsorbability of antibiotics onto the carrier is important when locally administered.

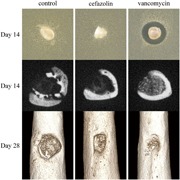

Osteomyelitis is an infection of the bone marrow that can progress to bone destruction. Although there is an ongoing development of therapeutic techniques, osteomyelitis remains one of the most challenging disorders for orthopedic physicians. A combined surgical and pharmaceutical approach has been the gold standard in clinical practice for the treatment of osteomyelitis.^1^ The main surgical therapy is radical debridement, and bone fenestration, bone augmentation, soft tissue salvaging or amputation is occasionally considered,^1^ while pharmacologically, 4–6 weeks of high‐dose antibiotic therapy are recommended.^2^, ^3^, ^4^, ^5^ The local administration of antibiotics with drug delivery systems is desirable in terms of achieving a sufficient drug concentration at local lesions and reducing systemic toxicity, yet difficulties in maintaining a long‐term pharmacologic effect and the impact of residual artificial materials in infection sites must be considered. For example, vancomycin‐impregnated cement is commonly implanted for the local administration of antibiotics in traumatic osteomyelitis in Japan,^6^ Additionally, bone regeneration is required for the treatment of osteomyelitis because of the associated osteolysis.^7^

To improve upon current delivery scaffolds, we utilized a porous hydroxyapatite/collagen composite (HAp/Col) as an artificial bone substitute with distinctive characteristics.^8^, ^9^, ^10^, ^11^ HAp/Col composite is composed of collagen fibers with HAp nanocrystal deposits, and the nanostructure resembles that of natural bone.^8^ HAp/Col has a sponge‐like elasticity that makes it easier to fit into bone defects and affords excellent biocompatibility and osteoconductivity. Furthermore, this porous HAp/Col (Refit®; HOYA Technosurgical Co., Tokyo, Japan) has been commercially available in Japan since 2013 for bone defects and has shown satisfactory bone regeneration clinically.^9^ Additionally, HAp/Col composite has a large surface area of 75 m^2^/g, and the nanoscale HA crystals enable it to absorb a large quantity of proteins and other molecules, such as drugs and ions.^12^

In our previous studies, the adsorbability of alendronate and bone morphogenetic protein 2 (BMP‐2) to HAp/Col was demonstrated.^13^, ^14^, ^15^ We have reported that the adsorbability of BMP‐2 onto HAp/Col enables long‐term release and enhances the bone formation in rat bone defect model.^15^

The objective of the present study is to investigate the feasibility of local antibiotic administration in the acute phase of a traumatic osteomyelitis model in rats using HAp/Col as a drug delivery system. Given that *Staphylococcus aureus* has been demonstrated clinically to be the most common causative pathogen for osteomyelitis,^16^ we will likewise examine *S. aureus* in our rat model of osteomyelitis. We hypothesized that higher adsorbability of antibiotics onto HAp/Col will result in more effective treatment and healing of acute osteomyelitis lesions, as measured by the number of bacteria, the extent of bone destruction, and bone regeneration.

## MATERIALS AND METHODS

### Animals

All animal experiments were performed after approval was obtained from the Animal Experiment Committee of Tokyo Medical and Dental University and Narita Animal Science Laboratory and were conducted according to the guidelines of our institution on the care and use of laboratory animals (A2017‐026A, 18L‐R008). Wistar rats were purchased from Sankyo Labo Service Corporation (Tokyo, Japan) and were acclimatized for 1 week prior to the experiment. Rats were housed in plastic cages with two animals to a cage at a constant temperature and humidity with a standard 12:12 h light/dark cycle. The animals were fed a standard laboratory chow and sterile water ad libitum. Euthanasia criteria included weight loss of 25%, weakness, pus discharge and/or fractures.

### Materials

Eight antibiotics frequently used in clinical practice were evaluated in this study: amikacin sulfate (014‐24941, Wako, Osaka, Japan; AMK), cefazolin sodium salt (CEZ; 038‐22981; Wako, Osaka, Japan), cefotiam hydrochloride (CTM; 029‐22911; Wako), daptomycin (DPT; 103060‐53‐3; Wako), minocycline hydrochloride (MINO; PAA105932; Pfizer, NY), piperacillin (PIPC; P3462; LKT Laboratories, MN), teicoplanin (TEIC; 1756‐100; Wako), and vancomycin hydrochloride (VCM; 226‐01301; Wako). The concentration of the antibiotic solution in porous HAp/Col for each animal experiment was generally decided assuming a systemic daily dose per 5 cm^3^ of porous HAp/Col and considering the solubility in saline (Table 1). The volume percentage of antibiotic solution that infiltrated into porous HAp/Col was 60%.

**Table 1:** The Amount of Impregnated Antibiotics Per 1 cm^3^ of Hydroxyapatite/Collagen (HAp/Col) Based on the General Systemic Daily Dose

	AMK	CEZ	CTM	DPT	MINO	PIPC	TEIC	VCM
General systemic daily dose (mg)	400	2,000	500	350	200	1,000	400	500
Impregnated amount per 1 cm^3^ HAp/Col (mg)	40	200	100	70	20	200	80	100

### Properties of Antibiotic Adsorption to HAp/Col

**Figure 1:**
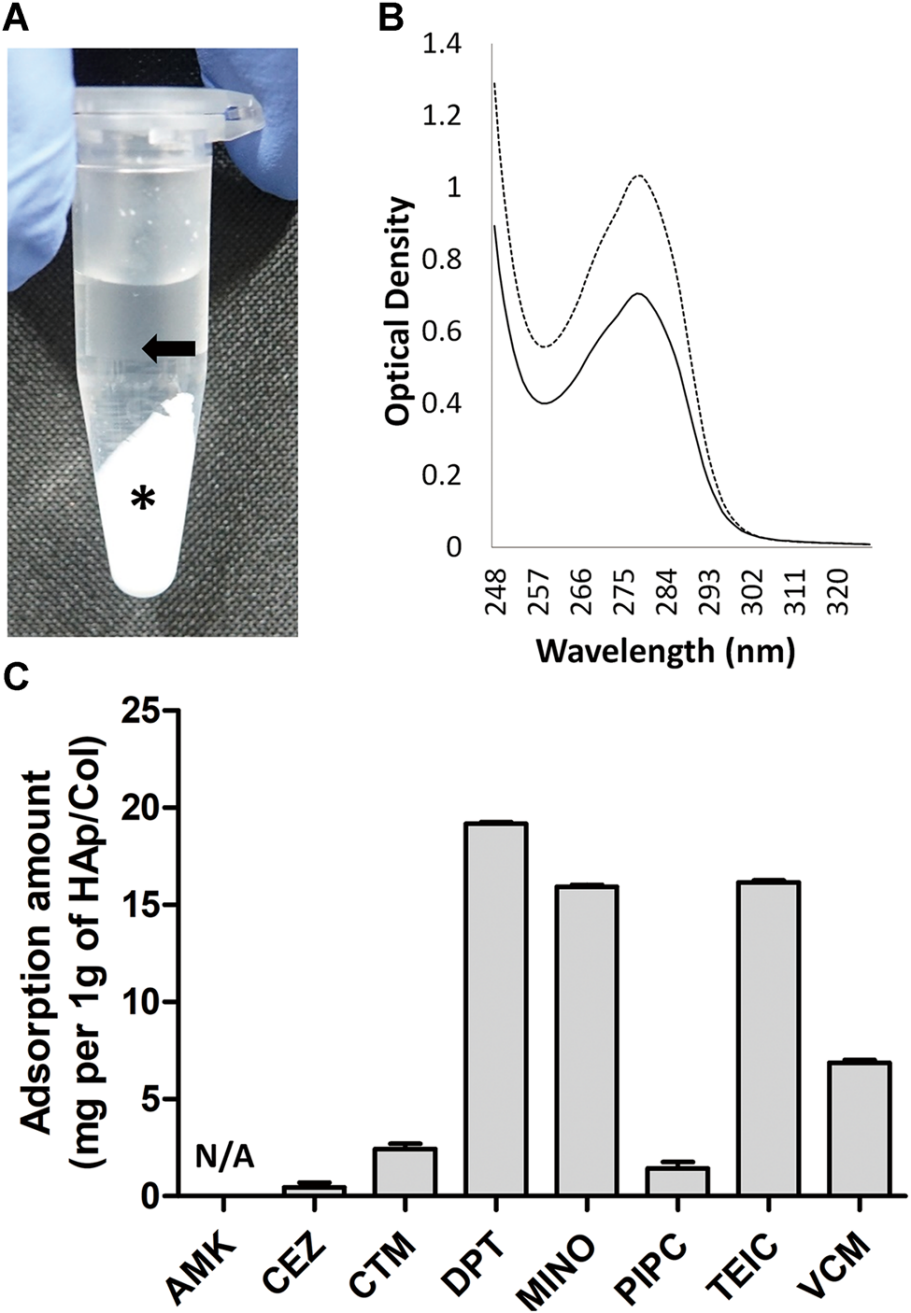
Adsorption of antibiotics to hydroxyapatite/collagen (HAp/Col). (A) A photograph of a mixture of HAp/Col (asterisk) and antibiotic solution (arrow) after centrifugation. (B) A representative graph of optical density (VCM). The difference between the reference solution (dotted line) and the mixture of antibiotic solution and HAp/Col (solid line) at the peak wavelength indicates the amount of adsorbed antibiotic. (C) The amount of adsorbed antibiotic (mg) per 1 g of HAp/Col. The optical density of AMK could not be detected. N/A, not available. [Color figure can be viewed at wileyonlinelibrary.com]

The antibiotic solutions (1.0 ml, 1.0 mg/ml) were mixed with 50 mg of HAp/Col fibers in 1.5 ml plastic tubes, which were shaken with horizontal rotation at 2 Hz for 24 h at 4°C and then centrifuged at 17,860 *g* for 5 min (Fig. 1A). The supernatant was collected and diluted in half with an equal volume of normal saline, and the optical density was measured. To prepare reference solutions of each antibiotic, the original antibiotic solutions (prior to incubation with HAp/Col) were mixed with the same volume of normal saline to compensate for the effect of phosphoric acid ions eluting from HAp/Col. The proportion of the peak against the reference solution of each antibiotic at specific wavelengths was calculated to determine the concentration of antibiotics in the supernatant, and the amount of adsorbed antibiotic was determined by subtracting this value from the original concentration (Fig. 1B).

### Longitudinal Antibacterial Effect of HAp/Col With Antibiotics After Implantation

Antibiotic solutions at the concentration described above infiltrated porous HAp/Col (4 × 4 × 4 mm) for 30 min before implantation (Table 1).

Wistar rats (40 males, 10 weeks old, 220–250 g) were anesthetized by isoflurane inhalation and given a subcutaneous injection of 2 mg/kg meloxicam as an analgesic. After the dorsum was shaved and disinfected, four 5‐mm longitudinal incisions were made bilaterally at the L1 and L5 levels 10 mm from the midline. One porous HAp/Col composite impregnated with antibiotics was implanted in the subcutaneous tissue via each incision, followed by skin closure with 4‐0 nylon. After surgery, the rats were able to move freely without any limitations in an SPF level cage. Five rats were assigned to each of the eight antibiotic groups and sacrificed at 1, 3, 5, 7, or 14 days after surgery (*n* = 1; four HAp/Col implants), and the porous HAp/Col implants were harvested and stored at −80°C.

One milliliter of bacterial solution containing 1 × 10^7^ colony‐forming units (CFU) of methicillin‐susceptible *S. aureus* subsp. aureus (MSSA, NCTC 13841) (Public Health England, Salisbury, UK) was diluted in 20 ml of agar medium (46°C) on a 10‐cm dish. The harvested HAp/Col composite was embedded in the center of the agar medium, followed by immediate congealing at room temperature. After a 24‐h incubation at 37°C, the inhibition zone appearing around the porous HAp/Col was measured with ImageJ software.^17^

### Implantation of HAp/Col With Antibiotics Into a Non‐Infected Femur

Wistar rats (60 males, 12 weeks old, 240–270 g) were anesthetized, and a 10‐mm longitudinal incision was made on the lateral epicondyle of the bilateral femur after the legs were shaved and disinfected. The femur was then approached through the intermuscular space between the biceps femoris and vastus lateralis muscles, and the lateral epicondyle was exposed. A ϕ3‐mm monocortical hole was made on the lateral epicondyle with a 2.0‐mm electrical drill (Fig. 3A), followed by flushing the bone dust with normal saline. The rats were assigned to one of the eight antibiotic groups or normal saline (NS) or control (no implant) group, and the porous HAp/Col (3 × 3 × 4 mm) containing the appropriate solution was implanted into the hole. The rats were sacrificed at 2, 4, and 8 weeks after surgery, and both femurs were harvested (*n* = 2; four femurs).

**Figure 2:**
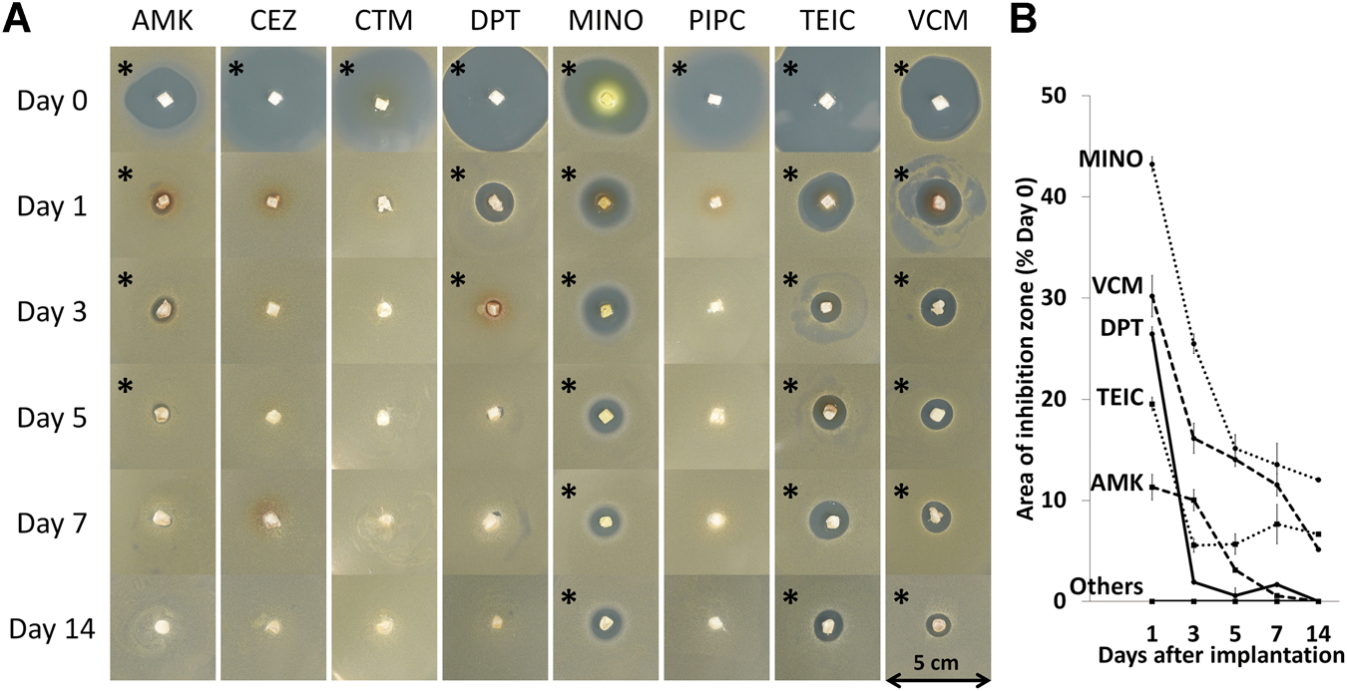
Longitudinal antibacterial effect of hydroxyapatite/collagen (HAp/Col) impregnated with antibiotics after subcutaneous implantation. (A) Representative photographs of culture dishes. The white squares are HAp/Col (5 × 5 × 5 mm), and the translucent circles are inhibition zones. The asterisks indicate the effective inhibition zone. (B) The area of the inhibition zone (percentage relative to day 0) on days 1, 3, 5, 7, and 14. MINO, TEIC, and VCM showed antibacterial effects, even at day 14. The other antibiotics include CEZ, CTM, and PIPC. [Color figure can be viewed at wileyonlinelibrary.com]

**Figure 3:**
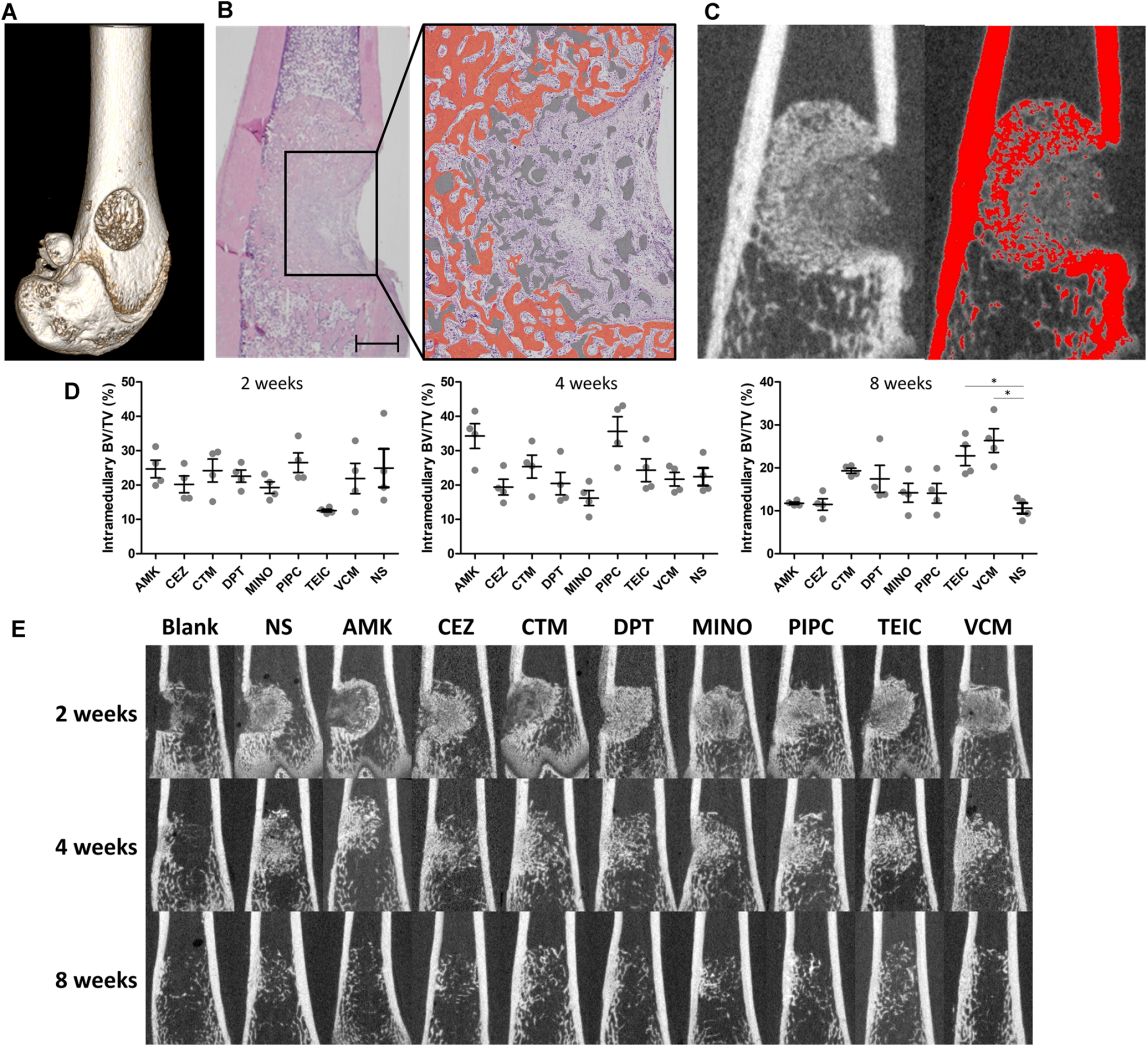
Implantation of hydroxyapatite/collagen (HAp/Col) with antibiotics into a non‐infected femur. (A) three‐dimensional computed tomography (CT) image of the bone hole at the lateral condyle. (B) Representative histological image at 2 weeks after implantation. The right schematic image shows newly formed bone and HAp/Col as orange and gray, respectively (scale bar: 1,000 µm). (C) CT scan of the same slice as in (B). The red area represents the area with a CT value of greater than 2,000, which was defined as bone in conjunction with the histological images. (D) BV/TV (%) in the intramedullary space measured by CT scan as the volume containing a CT value of greater than 2,000 at 2, 4, and 8 weeks after implantation. *p < 0.05 versus NS. (E) Representative coronal slices of CT scans in each antibiotic group. [Color figure can be viewed at wileyonlinelibrary.com]

A micro‐CT scan (CosmoScan GX, Rigaku Co., Tokyo, Japan; FOV 10 mm) of the specimens was performed, and bone regeneration was quantified with ImageJ.^17^ To distinguish newly formed bone from HAp/Col, the areas with a CT value above 2000 indicated bone density in conjunction with the histological findings (Fig. 3B and C). The femur was evaluated for 3 mm (150 slices) centered on the implantation site, and the bone volume/tissue volume ratio in the medullary space was calculated. After micro‐CT analysis, the femurs were decalcified with 10% EDTA and assessed histologically as paraffin‐embedded sections with hematoxylin and eosin (H&E) staining.

### Intramedullary Concentration of Antibiotics (CEZ and VCM) in the Femur

CEZ and VCM were selected for this experiment as representative antibiotics that were hardly or highly adsorbed to HAp/Col, respectively, in a previous experiment.

Wistar rats (6 males, 12 weeks old, 240–270 g) were assigned to the CEZ or VCM group (*n* = 3); porous HAp/Col (3 × 3 × 4 mm) with antibiotics was implanted into the hole in the right femur as described for the previous experiment. The femurs were harvested at 1 week after surgery, and a 10‐mm section of the diaphysis centered on the implanted hole in the center was cut. Then, the medullary space of the diaphysis was washed five times with 1 ml of saline using a 23G needle. After centrifugation, the antibiotic concentration was measured in the supernatant.

A series of standard solutions of known concentrations of VCM were injected into a CAPCELL‐PAK C18 UG120 column (5 μm, ϕ4.6 mm × 25 cm; Shiseido, Tokyo, Japan) of an Elite LaChrom high‐performance liquid chromatography (HPLC) system (Hitachi High‐Technologies, Tokyo, Japan). The HPLC conditions were as follows: column temperature, 30°C; mobile phase A, triethylamine buffer (pH 3.2)/acetonitrile/tetrahydrofuran = 92/7/1 (v/v); isocratic elution, phase A (20 min); flow rate, 1 ml/min; wavelength, 280 nm; and injection volume, 20 μl. The peak area of VCM in each standard solution was measured and plotted against VCM concentration to generate a calibration curve. The VCM concentration in each test sample was determined under the same conditions. The CEZ concentration in each test sample was determined by HPLC under the following conditions: column temperature, 25°C; mobile phase B, 15% acetonitrile with 6.6 mM disodium hydrogen phosphate 12‐hydrate and 2.2 mM citric acid monohydrate; isocratic elution, phase B (20 min); flow rate, 1 ml/min; wavelength, 254 nm; and injection volume, 20 μl. The other conditions were the same as for VCM.

### The Possible Inactivation Effect of HAp/Col on Antibiotics (CEZ and VCM)

Antibiotic solutions (CEZ and VCM) at the indicated concentration (Table 1) infiltrated the porous HAp/Col (3 × 3 × 4 mm), which was then crushed in 1 ml of saline. After centrifugation, the concentration of antibiotics in the supernatant was measured. New antibiotic solutions with a calculated concentration were prepared, and then, a ϕ5‐mm disc of filter paper was infiltrated with the supernatant or antibiotic solution. The disc was put on the center of a 10‐cm dish that contained MSSA‐distributed agar medium as described above. After a 24‐h incubation at 37°C, the diameter of the inhibition zone around the disc was measured (*n* = 6).

### Release Kinetics of VCM From Impregnated HAp/Col In Vitro

VCM solutions (1.0 ml, 1.0 mg/ml) were mixed with 50 mg of HAp/Col fibers in 1.5‐ml plastic tubes, which were shaken horizontally at 2 Hz for 24 h at 4°C and then centrifuged. After the supernatant was removed, the HAp/Col was washed with normal saline. After centrifugation, the release experiment of impregnated HAp/Col started with 1.0 ml of normal saline with horizontal rotation at 2 Hz for 24 h at 4°C. At 2, 24, 48, 72, and 120 h of incubation, the supernatant was collected, and the optical density was measured using the same method described above (*n* = 3). The released amount of antibiotics was determined by multiplying the solution volume (1.0 ml) and the concentration calculated with measured optical density and standard curve.

### Acute Osteomyelitis Model (CEZ and VCM)

CEZ and VCM were also evaluated in this experiment. MSSA was used for this experiment instead of MRSA to assess the effect of adsorbability on antibacterial activity while eliminating the factor of sensitivity to specific antibiotics.

A total of 54 Wistar rats (male, 12 weeks old, 240–270 g) were examined. The anesthesia, skin incision and surgical approach were the same as stated above. Then, a ϕ1‐mm monocortical hole was made on the lateral side of both femurs, 2 mm proximal from the lateral articular capsule to avoid arthritis, followed by inoculation with an MSSA solution (1 × 10^3^ CFU/2 μl) with a micropipette. The hole was then sealed with wax.

**Figure 4:**
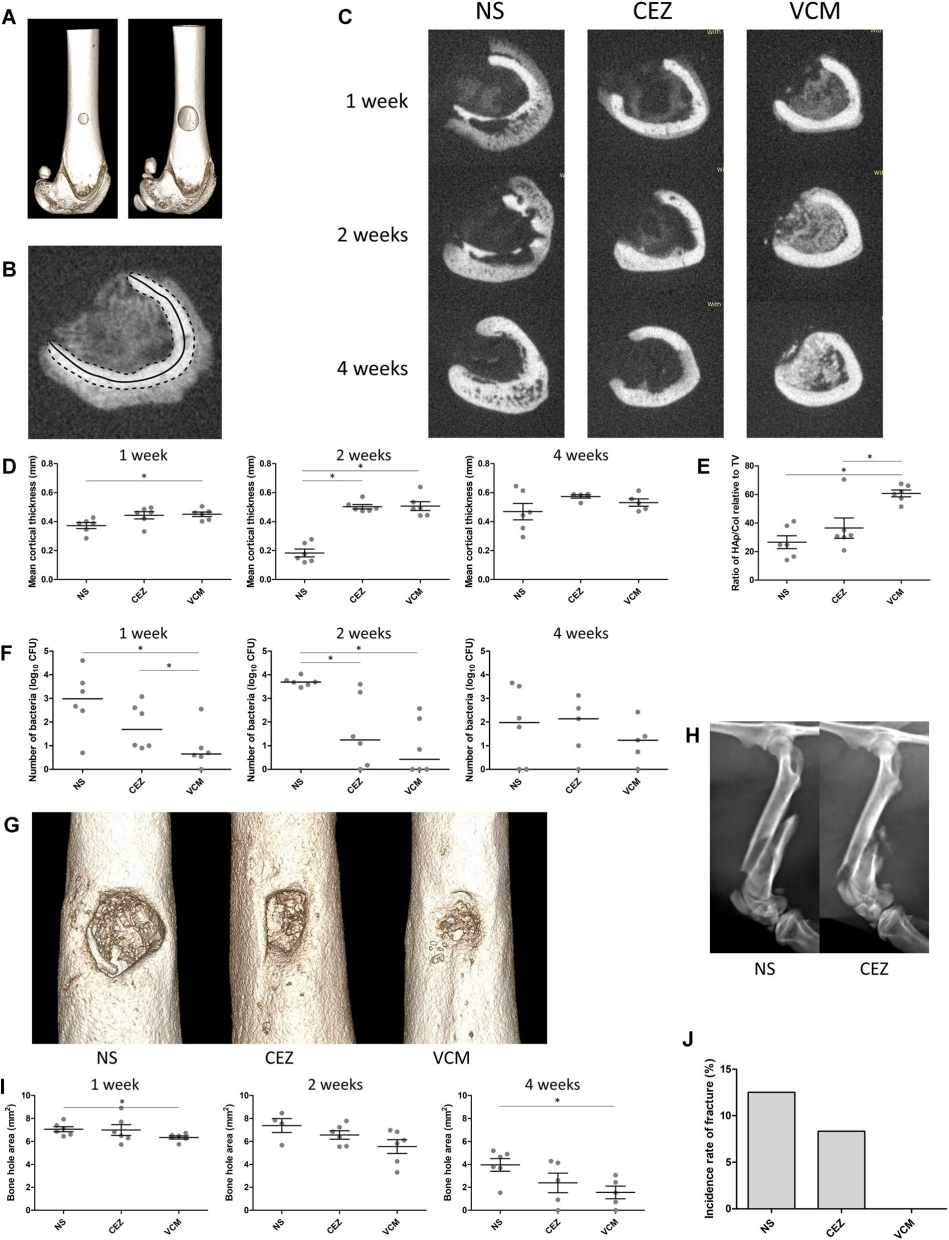
Therapeutic effect of hydroxyapatite/collagen (HAp/Col) impregnated with CEZ or VCM in the acute osteomyelitis model. (A) Three‐dimensional (3D) computed tomography (CT) image of the bone hole. A ϕ1‐mm hole (left image) was made to inoculate MSSA, and the hole was dilated to ϕ3 mm (right image) 7 days after inoculation. (B) An axial slice of the CT scan. The mean cortical thickness was obtained by dividing the cortical area (dotted line) by the cortical length (solid line). (C) Representative axial slices of the CT scan at 1, 2, and 4 weeks after implantation in the NS, CEZ, and VCM groups. (D) Mean cortical thickness at 1, 2, and 4 weeks after implantation. *p < 0.05. (E) The ratio of HAp/Col volume remaining relative to intramedullary tissue volume at 1 week after implantation. *p < 0.05. (F) MSSA count after a 24‐h incubation of intramedullary wash solution (50 μl) on agar medium. *p < 0.05. (G) 3D CT reconstruction of bone holes at 4 weeks after implantation. (H) X‐ray photographs of fracture cases. (I) Bone hole area at 1, 2, and 4 weeks after implantation. *p < 0.05. (J) Fracture incidence rate among 24 operated femurs. [Color figure can be viewed at wileyonlinelibrary.com]

One week after surgery, the rats underwent implantation surgery. The ϕ1‐mm hole made in the first surgery was dilated to ϕ3 mm and flushed with 1 ml of saline to remove bone fragments (Fig. 4A). The rats were assigned to the NS, CEZ, or VCM group (54 total rats), and porous HAp/Col (3 × 3 × 4 mm) with antibiotics or normal saline was implanted into the dilated hole without debridement. The rats were sacrificed at 1, 2, and 4 weeks after surgery (*n* = 6), and both femurs were harvested.

The harvested right femur was immediately fixed with 4% paraformaldehyde and evaluated by micro‐CT scanning. Cortical bone destruction, the volume of remaining HAp/Col and the size of the bone hole were quantified with ImageJ.^17^ Cortical bone destruction was calculated as the mean cortical thickness in the axial slice with the most cortical bone destruction in each individual. The mean cortical thickness was calculated by dividing the original cortical area by the cortical length (Fig. 4B). After micro‐CT analysis, the femurs were assessed histologically with H&E staining as described above.

The left femur was used for the quantitative analysis of bacteria. Immediately after harvest, a 15‐mm‐long cylindrical section of the femur diaphysis centered on the implanted hole was cut. Then, the medullary cavity was thoroughly scraped using a 23G needle to remove trabecular bone and washed five times with 10 ml of phosphate‐buffered saline (PBS). The washes were collected and diluted 1×, 10×, 100×, and 1,000× with PBS, and 50 μl of each solution was spread on agar medium in the petri dish. To eliminate the effect of possible remaining antibacterial activity on the CFU count, the volume of the medullary cavity (approximately 50 mm^3^) was diluted 1:200,000 at the maximum when used for culture; thus, the concentration of antibiotics should be sufficiently lower than the MIC value. After a 24‐h incubation at 37°C, the CFU value was determined, and the average of the duplicates at the optimal concentration.

### Statistical Analysis

The data obtained from each group were averaged, and the results are presented as the mean and standard deviation. Statistical analyses were conducted using SPSS 24 (IBM, NY). In each experiment, the overall differences between groups were determined by one‐way analysis of variance, followed by multiple comparison testing using the Tukey–Kramer test or Mann–Whitney test (for the MSSA count in the osteomyelitis model). Fischer's exact test was also used for the MSSA count in the osteomyelitis model. The differences were considered statistically significant at *p* < 0.05.

## RESULTS

### Antibiotic Adsorption to HAp/Col

DPT (19.2 ± 0.2 mg), MINO (15.9 ± 0.2 mg), TEIC (16.1 ± 0.2 mg), and VCM (6.9 ± 0.3 mg) were highly adsorbed to HAp/Col, whereas CEZ (0.4 ± 0.6 mg), CTM (2.4 ± 0.6 mg), and PIPC (1.4 ± 0.7 mg) were hardly adsorbed (results presented as the mean adsorbed amount per 1 g of HAp/Col) (Fig. 1C). The concentration of AMK could not be measured because the corresponding samples showed no optical density in the instrument measurement range.

### **Longitudinal Antibacterial Effect of HAp/Col With Antibiotics** A**fter Implantation**

HAp/Col infiltrated with antibiotics was implanted into the subcutaneous tissue, not bone, to avoid implant absorption by osteoclasts and to exclude the restriction of drug effusion by surrounding dense cortical bone.

Although all the antibiotics showed an inhibition zone at day 0 (prior to implant), CEZ, CTM, and PIPC groups lost activity only 24 h after implantation (Fig. 2A). On the other hand, antibacterial activity was maintained through 14 days after implantation in the TEIC, VCM, and MINO groups, and the mean ratio of the inhibition zone area at day 14 compared with day 0 was 6.7 ± 4.0%, 5.1 ± 2.2%, and 12.0 ± 4.2%, respectively. AMK and DPT generated an inhibition zone for up to 7 days (Fig. 2B).

This result, combined with that of the prior experiment on adsorption property, indicated that adsorbability to HAp/Col is likely necessary for a prolonged antibacterial effect. Additionally, the shape of the inhibition zone seen in the culture was almost a perfect circle, which indicated that the antibiotics were homogeneously distributed throughout the HAp/Col.

### The Effect of HAp/Col Impregnated With Antibiotics on Bone Regeneration

As a control, intramedullary BV/TV at 2, 4, and 8 weeks after implantation in the NS group was 25.0 ± 11.2, 22.4 ± 5.1, and 10.6 ± 2.5, respectively. Compared with the NS group, all the antibiotic groups showed no significant decrease in BV/TV at all observation points. BV/TV significantly increased in the TEIC (22.8 ± 4.6) and VCM groups (26.4 ± 5.6) at 8 weeks compared with the NS group (Fig. 3D and E).

The histological analysis showed intramedullary bone formation along the column of porous HAp/Col, mainly near the periphery, at 2 weeks, and in general, about half of the HAp/Col remained. At 4 weeks, HAp/Col was almost completely absorbed and replaced by newly formed bone. The bone volume was highest at this point in all groups. At 8 weeks, HAp/Col had completely disappeared, and the formed bone was remodeled.

### Intramedullary Concentration of Antibiotics (CEZ and VCM) in the Rat Femur at 1 Week After Implantation

At 1 week after implantation, the concentration of antibiotics in the intramedullary wash was evaluable in the VCM group (2.89, 0.82, and 5.88 µg/ml, *n* = 3), whereas CEZ was not detected in any group. The intramedullary VCM concentration calculated relative to intramedullary space obtained by CT analysis was 59.0, 13.9, and 95.2 µg/ml (Table 2).

**Table 2:** Concentration of CEZ and VCM in the Intramedullary Space at 7 Days After Implantation

Concentration of Antibiotics (µg/ml)	Intramedullary Wash	Intramedullary Space
CEZ (*n* = 3)	0	0
	0	0
	0	0
VCM (*n* = 3)	2.9	59.0
	0.8	13.9
	5.9	95.2

### Possible Inactivation Effect of HAp/Col on Antibiotics (CEZ and VCM)

The diameter of the inhibition zone was 19.3 ± 0.8 mm (VCM with HAp/Col), 17.9 ± 0.3 mm (VCM alone), 19.1 ± 0.5 mm (CEZ with HAp/Col), and 19.2 ± 0.4 mm (CEZ alone). There were no significant differences between groups with or without HAp/Col. These results did not indicate any possible antibiotic inactivation effect of HAp/Col.

### Release Kinetics of VCM From Impregnated HAp/Col In Vitro

The concentrations of VCM in the solution at 2, 24, 48, 72, and 120 h were 42 ± 4.0, 110 ± 1.6, 114 ± 12.3, 141 ± 14.7, and 124 ± 16.9 mg/ml, respectively. Given that HAp/Col adsorbed approximately 345 mg of VCM per 50 mg of HAp/Col in the adsorption experiment, one‐third of the adsorbed VCM was released within the first 24 h, and the remaining VCM seemed to be associated with the HAp/Col for at least 120 h. It should be noted that the degradation of HAp/Col in vivo might accelerate the release of VCM.

### Treatment Effect of Porous HAp/Col Containing Antibiotics (CEZ and VCM) in an Acute Osteomyelitis Rat Model

The femurs inoculated with *S. aureus* showed aggressive destruction in the group implanted with HAp/Col and saline (Fig. 4C). The mean cortical thicknesses at the axial slice with the greatest destruction was 0.37 ± 0.05 mm (1 week), 0.18 ± 0.07 mm (2 weeks), and 0.47 ± 0.14 (4 weeks) (Fig. 4D). At 1 week after implantation, the VCM group (0.45 ± 0.04 mm) showed a significant decrease in cortical destruction compared with the NS group, and the same trend was seen in the CEZ group (0.44 ± 0.06 mm), though this difference was not statistically significant. At 2 weeks after implantation, both the VCM (0.51 ± 0.07 mm) and CEZ (0.50 ± 0.04 mm) groups showed a marked decrease in cortical bone destruction.

A CT value range of 800–2,000 was used as the density of HAp/Col, and the volume of HAp/Col remaining was calculated as the volume within this range in the medullary space. The HAp/Col was significantly maintained in the VCM group compared with the NS and CEZ groups at 1 week after implantation, and the ratio of HAp/Col volume to intramedullary space was 26.6 ± 11.1%, 36.5 ± 17.4, and 60.8 ± 5.9% (Fig. 4E).

The cultures of intramedullary wash in the NS group showed a marked increase in CFU at 1 week (10^3.0^ CFU; median) and 2 weeks (10^3.7^ CFU) after implantation and a decrease at 4 weeks after implantation (10^2.0^ CFU) (Fig. 4F). MSSA proliferation was significantly prevented at 2 weeks in the CEZ (10^1.24^ CFU) and at 1 and 2 weeks in VCM groups (10^0.65^ and 10^0.42^ CFU) (Fig. 4F). The MSSA count was lower in the VCM group than in the CEZ group at every time point, and there was a significant difference at 1 week.

To support these findings, Fisher's exact test (one‐tailed) was applied in which the cut off was determined as 10 CFU. Significant decrease was observed in VCM group at 1 (*p* = 0.04) and 2 weeks (*p* = 0.03) compared with NS group, while no difference was seen between CEZ and NS groups.

The bone hole area was significantly decreased in the VCM group at 4 weeks after implantation compared with the NS group (Fig. 4G and I). The same trend was noted in the CEZ group, but the difference was not statistically significant from the NS group.

**Figure 5:**
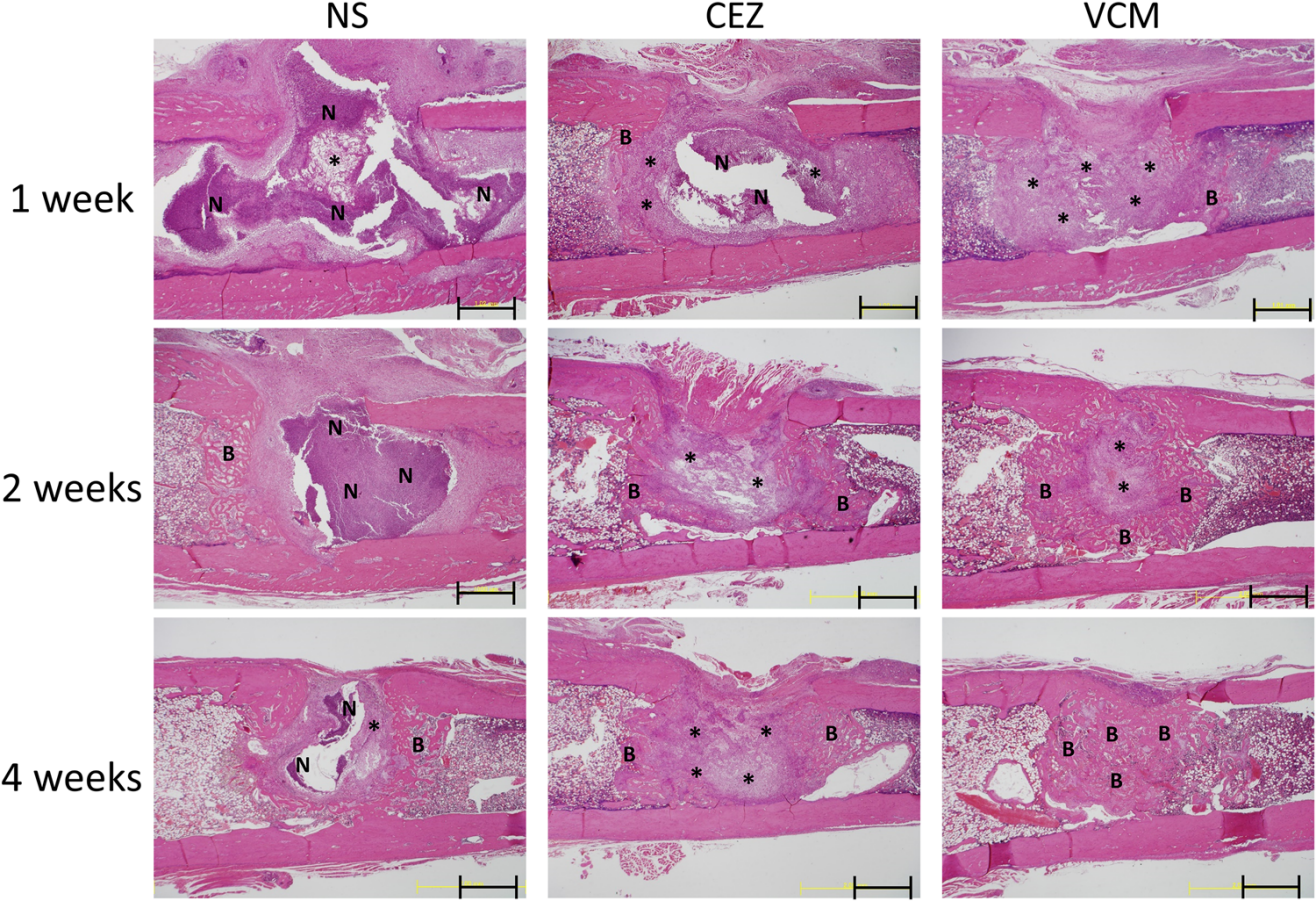
Histological images of the implant site (hematoxylin and eosin staining). Images were obtained 1, 2, and 4 weeks after the implantation of hydroxyapatite/collagen (HAp/Col) impregnated with NS, NEZ, or VCM in the acute osteomyelitis model (scale bar: 1,000 µm). A greater amount of neutrophil (N) infiltration was observed in the NS group than in the other groups. Bone regeneration (B) was the greatest in the VCM group at 2 and 4 weeks, and HAp/Col was maintained in the VCM group at 1 week. (Asterisk; HAp/Col). [Color figure can be viewed at wileyonlinelibrary.com]

Histologically, there was greater neutrophil infiltration at 1 week in the NS group while that was limited in VCM group (Fig. 5). Implanted HAp/Col was generally degraded in the NS and CEZ group, but comparatively maintained in the VCM group. At 2 weeks, neutrophil infiltration increased in the NS group but that was limited in the CEZ and VCM groups. Bone regeneration was the largest in VCM group especially at 2 and 4 weeks, and HAp/Col was almost replaced with newly formed bone at 4 weeks in VCM group.

In this series, although there were three and two cases of femur fracture in the NS and CEZ groups, respectively, there were none in the VCM group (Fig. 4H); the animals with femur fractures were immediately sacrificed after fracture detection, and the fractured femurs were excluded from the following bacterial and CT assessments. All the fractures were seen at 2 weeks after implantation, so the rate of fracture of the 24 femurs with implants (for cases observed for at least 2 weeks) was 12.5% in the NS group and 8.3% in the CEZ group (Fig. 4J).

## DISCUSSION

DPT, MINO, TEIC, and VCM showed high adsorbability to HAp/Col. Of these antibiotics, MINO, TEIC, and VCM had antibacterial activity for at least 14 days after subcutaneous implantation with HAp/Col. MINO is known to chelate calcium phosphate, potentially accounting for its high adsorption onto HAp/Col.^18^ The electrostatic energy of HAp was reported to play a role in the interaction between drugs and the HAp surface.^19^ Given that the surface of c plane of HAp is negatively charged, cationic VCM can adsorb onto HAp^20^ because the amino groups of the VCM are protonated.^21^ As TEIC resembles the structure as VCM as they both are glycopeptide antibiotics, charge‐based interactions may likewise account for TEIC high adsorbability to HAp.^22^ DPT, which adsorbed to HAp/Col, did not generate an inhibition zone beyond 1 day after implantation. This finding might be due to DPT degradation under the in vivo conditions because DPT underwent longitudinal degradation when incubated in FBS at 37°C (data not shown). These results suggest that long‐term antibacterial activity requires both adsorbability to HAp/Col and in vivo drug stability. However, future study with other materials that do not adsorb antibiotics should be required to eliminate the possibility that inherent properties of antibiotics themselves caused these results.

The actual intramedullary concentration of VCM was 13.9–95.2 µg/ml. The Clinical Laboratory Standards Institute defined MRSA as sensitive to VCM as the minimum inhibitory concentration is less than 2.0 µg/ml^23^; thus, theoretically, HAp/Col impregnated with VCM should have antibacterial activity for at least 7 days after implantation.

We selected CEZ and VCM in the treatment experiment for the acute osteomyelitis model because they represent antibiotics that can be hardly or highly adsorbed to HAp/Col, and compared with the CEZ group, the VCM group showed greater prevention of HAp/Col degradation, decreased fracture incidence and accelerated decrease in the bone hole area. A significant difference in the CFU of MSSA was not seen after 4 weeks between the NS and CEZ/VCM groups because this acute osteomyelitis model was naturally curable and was not followed by a chronic condition. Additionally, gentamycin, which is commonly administered locally in Europe, was not included in this study because its optical density was hard to detect.

Polymethylmethacrylate cement is used as an antibiotic delivery vehicle, but this material must be subsequently removed.^24^, ^25^ Biodegradable antibiotic‐loaded calcium sulfate is also commercially available for the treatment of osteomyelitis.^26^ However, Ferguson reported a 4.6% fracture rate, which suggests that early bone regeneration at the bone infection site might be inadequate with calcium sulfate.^26^ On the other hand, the osteoconductivity of porous HAp/Col seems to be much higher than that of calcium sulfate, as indicated by experiments of HAp/Col implanted into non‐infected bones.^11^, ^27^, ^28^, ^29^ These results indicate that HAp/Col may have advantages in terms of early bone regeneration.

The present study was limited because systemic antibiotics were not compared, and there was no negative control for biomaterial implantation for the osteomyelitis model; we used HAp/Col without antibiotics as the negative control. Further studies should be performed to clarify these points prior to clinical application, and sensitivity and resistance to the antibiotics should be considered. Another limitation was that bacteria in muscle or cortical bone were not investigated; only trabecular bone was evaluated.

## CONCLUSION

The present study showed the longitudinal antibacterial activity of MINO, TEIC, and VCM implanted subcutaneously for up to 14 days owing to their adsorbability to HAp/Col composites. As a result of longitudinal antibacterial activity due to its adsorbability to HAp/Col, implantation of HAp/Col impregnated with VCM had a greater therapeutic effect than CEZ in terms of the number of bacteria, bone destruction, and bone regeneration. These results indicated that the adsorbability of antibiotics onto their carrier is important when locally administered and that HAp/Col might be a useful antibiotic delivery system for osteomyelitis.

## AUTHORS' CONTRIBUTION

S.E.: writing the paper and conducting all the experiments; K.H. and R.M.: supporting the animal experiments; T.Y., M.Y., and A.O.: analyzing the results; K.S.: preparing the Hap/Col and measuring the antibiotic concentrations. S.S.: Supporting the animal experiments and designing the experiments. All the authors have read the manuscript and have approved this submission.
